# Supplementary material for: Humanization of Pan-HLA-DR mAb 44H10 Hinges on Critical Residues in the Antibody Framework
Source: Antibodies (Basel). 2024 Jul 16;13(3):57. doi: 10.3390/antib13030057 (PMC11270187; doi:10.3390/antib13030057)
Supplement: Supplementary file 1 [file antibodies-13-00057-s001.zip › antibodies-3044178-supplementary.pdf]

Supplementary Tables

Table S1. Gene usages of humanized 44H10 candidates generated by IMGT CDR-grafting.

VERSION #	CDR DEFINITION	*IGHV*	*IGKV*
**1**	IMGT	4-59	1-16
**2**	IMGT	4-59	1-39
**3**	IMGT	4-59	1-6
**4**	IMGT	2-26	1-16
**5**	IMGT	2-26	1-39
**6**	IMGT	2-26	1-6
**7**	IMGT	4-4	1-16
**8**	IMGT	4-4	1-39
**9**	IMGT	4-4	1-6

Table S2. Gene usages of humanized 44H10 candidates generated by Kabat CDR-grafting.

VERSION #	CDR DEFINITION	*IGHV*	*IGKV*
**10**	Kabat	4-59	1-6
**11**	Kabat	4-59	1-16
**12**	Kabat	4-59	1-16
**13**	Kabat	4-59	1-6
**14**	Kabat	4-59	1-16
**15**	Kabat	4-59	1-16
**16**	Kabat	2-26	1-6
**17**	Kabat	2-26	1-16
**18**	Kabat	2-26	1-16

Table S3. X-ray crystallography data collection and refinement statistics for structures reported in this work (provided in separate file because of size considerations).

Table S4. Binding affinity and kinetics of humanized 44H10 antibodies to recombinant HLA-DR measured using biolayer interferometry. Measured on and off rates, as well as calculated dissociation constants are given, along with the standard error values.

		**Parental IgG**	**V17 IgG**	**V21 IgG**	**V22 IgG**
**HLA-DR**	**$K_{D\ apparent}$ (M)**	2.23E-09	N/A	9.46E-08	2.91E-08
	**$K_{D\ apparent}$ Error**	3.91E-11	N/A	1.82E-09	3.74E-10
	**$k_{on\ apparent}$ ($M^{-1}s^{-1}$)**	6.07E+05	N/A	1.59E+05	2.20E+05
	**$k_{on\ apparent}$ Error**	5.92E+03	N/A	2.98E+03	2.50E+03
	**$k_{off\ apparent}$ (s^{-1})**	1.35E-03	N/A	1.50E-02	6.39E-03
	**$k_{off\ apparent}$ Error**	1.97E-05	N/A	6.73E-05	3.88E-05

Table S5. Binding affinity and kinetics of wild-type (WT) and mutant V14 and V21 Fabs to recombinant HLA-DR measured using biolayer interferometry. Measured on and off rates, as well as calculated dissociation constants are given, along with the standard error values.

		V14 Fabs				V21 Fabs			
		WT	V71K	F78V	V71K + F78V	WT	K71V	V78F	K71V + V78F
HLA-DR	K_D (M)	N/A	N/A	N/A	2.06E-07	1.57E-07	N/A	N/A	N/A
	K_D Error	N/A	N/A	N/A	1.90E-09	1.49E-09	N/A	N/A	N/A
	k_{on} ($M^{-1}s^{-1}$)	N/A	N/A	N/A	1.99E+04	4.02E+04	N/A	N/A	N/A
	k_{on} Error	N/A	N/A	N/A	1.72E+02	3.58E+02	N/A	N/A	N/A
	k_{off} (s^{-1})	N/A	N/A	N/A	4.10E-03	6.32E-03	N/A	N/A	N/A
	k_{off} Error	N/A	N/A	N/A	1.32E-05	2.06E-05	N/A	N/A	N/A

Table S6. Binding affinity and kinetics of parental and humanized (V22) 44H10 IgGs to recombinant HLA-DR measured using biolayer interferometry. Measured on and off rates, as well as calculated dissociation constants are given, along with the standard error values.

	Storage Temp.	Timepoint	K_D (M)	K_D Error	k_{on} ($M^{-1}s^{-1}$)	k_{on} Error	k_{off} (1/s)	k_{off} Error
Parental IgG	Baseline		2.27E-07	7.79E-09	3.97E+05	1.30E+04	9.01E-02	9.45E-04
	-20°C	Week 1	4.13E-07	8.01E-09	3.01E+05	5.65E+03	1.24E-01	5.98E-04
		Week 2	3.29E-07	1.06E-08	3.82E+05	1.18E+04	1.26E-01	1.09E-03
		Week 3	2.96E-07	1.12E-08	4.09E+05	1.49E+04	1.21E-01	1.28E-03
		Week 4	3.33E-07	9.92E-09	3.54E+05	1.01E+04	1.18E-01	9.46E-04
	4°C	Week 1	2.45E-07	4.84E-09	3.85E+05	7.25E+03	9.44E-02	5.63E-04
		Week 2	2.07E-07	7.76E-09	4.85E+05	1.73E+04	1.00E-01	1.18E-03
		Week 3	1.91E-07	7.40E-09	5.06E+05	1.86E+04	9.67E-02	1.18E-03
		Week 4	2.03E-07	6.80E-09	4.63E+05	1.47E+04	9.38E-02	9.88E-04
	40°C	Week 1	2.97E-07	1.02E-08	3.75E+05	1.24E+04	1.12E-01	1.07E-03
		Week 2	2.91E-07	1.26E-08	4.00E+05	1.67E+04	1.16E-01	1.41E-03
		Week 3	2.75E-07	9.40E-09	3.85E+05	1.26E+04	1.06E-01	1.02E-03
		Week 4	2.89E-07	8.72E-09	3.47E+05	1.01E+04	1.00E-01	8.43E-04
Humanized (V22) IgG	Baseline		2.85E-07	1.97E-08	4.49E+05	2.95E+04	1.28E-01	2.76E-03
	-20°C	Week 1	4.32E-07	5.57E-08	3.80E+05	4.74E+04	1.64E-01	5.52E-03
		Week 2	3.25E-07	4.49E-08	4.23E+05	5.60E+04	1.37E-01	5.40E-03
		Week 3	4.14E-07	4.75E-08	3.24E+05	3.58E+04	1.34E-01	4.17E-03
		Week 4	3.93E-07	5.02E-08	3.34E+05	4.11E+04	1.31E-01	4.53E-03
	4°C	Week 1	3.01E-07	2.83E-08	4.08E+05	3.64E+04	1.23E-01	3.57E-03
		Week 2	2.92E-07	3.05E-08	4.68E+05	4.65E+04	1.37E-01	4.45E-03
		Week 3	2.62E-07	1.86E-08	4.33E+05	2.92E+04	1.14E-01	2.54E-03
		Week 4	2.12E-07	1.95E-08	4.70E+05	4.10E+04	9.96E-02	2.98E-03
	40°C	Week 1	7.44E-07	2.66E-08	2.31E+05	8.08E+03	1.72E-01	1.31E-03
		Week 2	3.27E-07	5.35E-08	4.31E+05	6.77E+04	1.41E-01	6.55E-03
		Week 3	2.60E-07	2.54E-08	4.17E+05	3.87E+04	1.08E-01	3.36E-03
		Week 4	4.03E-07	5.28E-08	3.45E+05	4.35E+04	1.39E-01	5.05E-03

Table S7. Table of contacts between the V22 Fab and HLA-DR.

V22 RESIDUE	HYDROPHOBIC INTERACTIONS	HYDROGEN BONDS
**H-TYR96**	α-Glu88	α-Val89 (x2)
**H-TYR99**	α-Asp171	α-Asp171
**H-HIS100A**	α-Pro86	
**H-TYR100B**	α-Pro86, A-Leu170	α-Pro87
**L-TYR32**	α-Thr83	α-Asn84 (x2)
**L-TYR49**		α-Asp142
**L-ALA50**	α-Val85	
**L-SER52**		β-Glu63
**L-THR53**	α-Leu144	
**L-LYS60**		β-Arg58, β-Glu65, β-Arg68
**L-SER65**		β-Glu81

Supplementary Figures

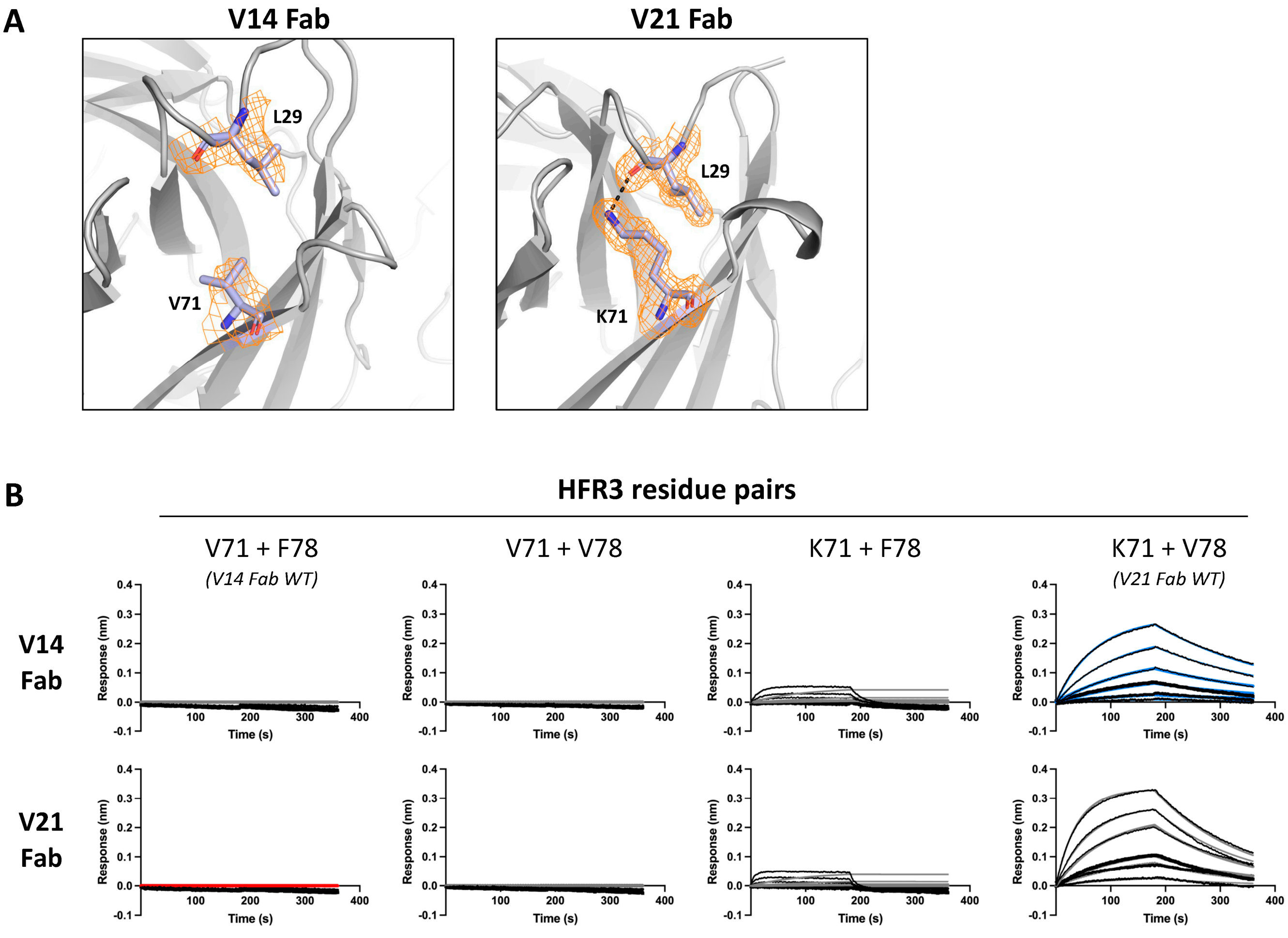

Figure S1. HFR3 residues 71 and 78 mediate humanized 44H10 binding to HLA-DR. (A) Composite omit map electron densities associated with residues at heavy chain positions 29 and 71 are shown as orange mesh and contoured at 1.0 σ. The hydrogen bond formed by K71 and L29 in the V21 Fab is indicated by a black dotted line. (B) BLI binding profiles of V14 and V21 wild-type, single mutant and double mutant Fabs to recombinant HLA-DR, where black lines represent measured binding and colored curves correspond to the data fitted to a 1:1 binding model.

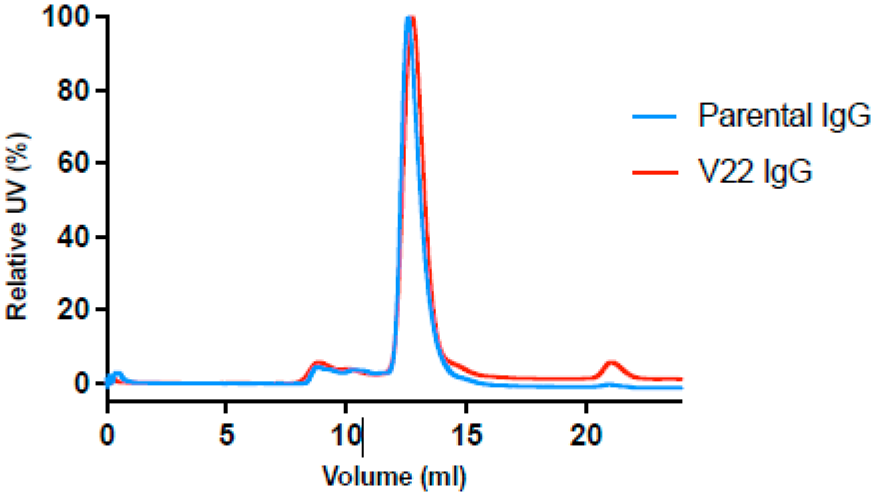

Figure S2. Purified parental and V22 IgGs are highly monodisperse. Size exclusion chromatography profiles (Superdex200) of parental 44H10 and V22 IgGs, revealing high monodispersity for both antibodies.

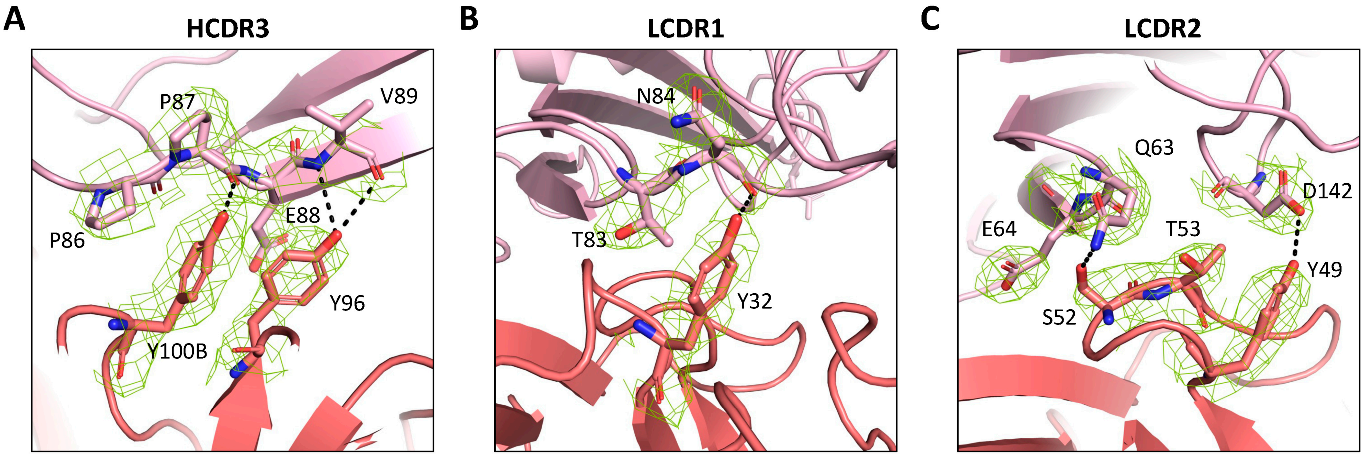

Figure S3. V22 Fab replicates key antigen contacts mediated by 44H10. (A-C) Composite omit map electron densities associated with residues in the V22 Fab HCDR3 (A), LCDR1 (B) or LCDR2 (C) that mediate key contacts with HLA-DR, shown as green mesh and contoured at 1.0 σ. Hydrogen bonds are indicated by black dotted lines.
